# Supplementary material for: Splice-Junction-Based Mapping of Alternative Isoforms in the Human Proteome
Source: Cell Rep. Author manuscript; Available in PMC 2020 Jan 15. (PMC6961840; doi:10.1016/j.celrep.2019.11.026)
Supplement: 3 [file NIHMS1546469-supplement-3.zip › DF2/PXD0010154/Spleen-116-Q32MZ4-EIKDSLAEVEEK.pdf]

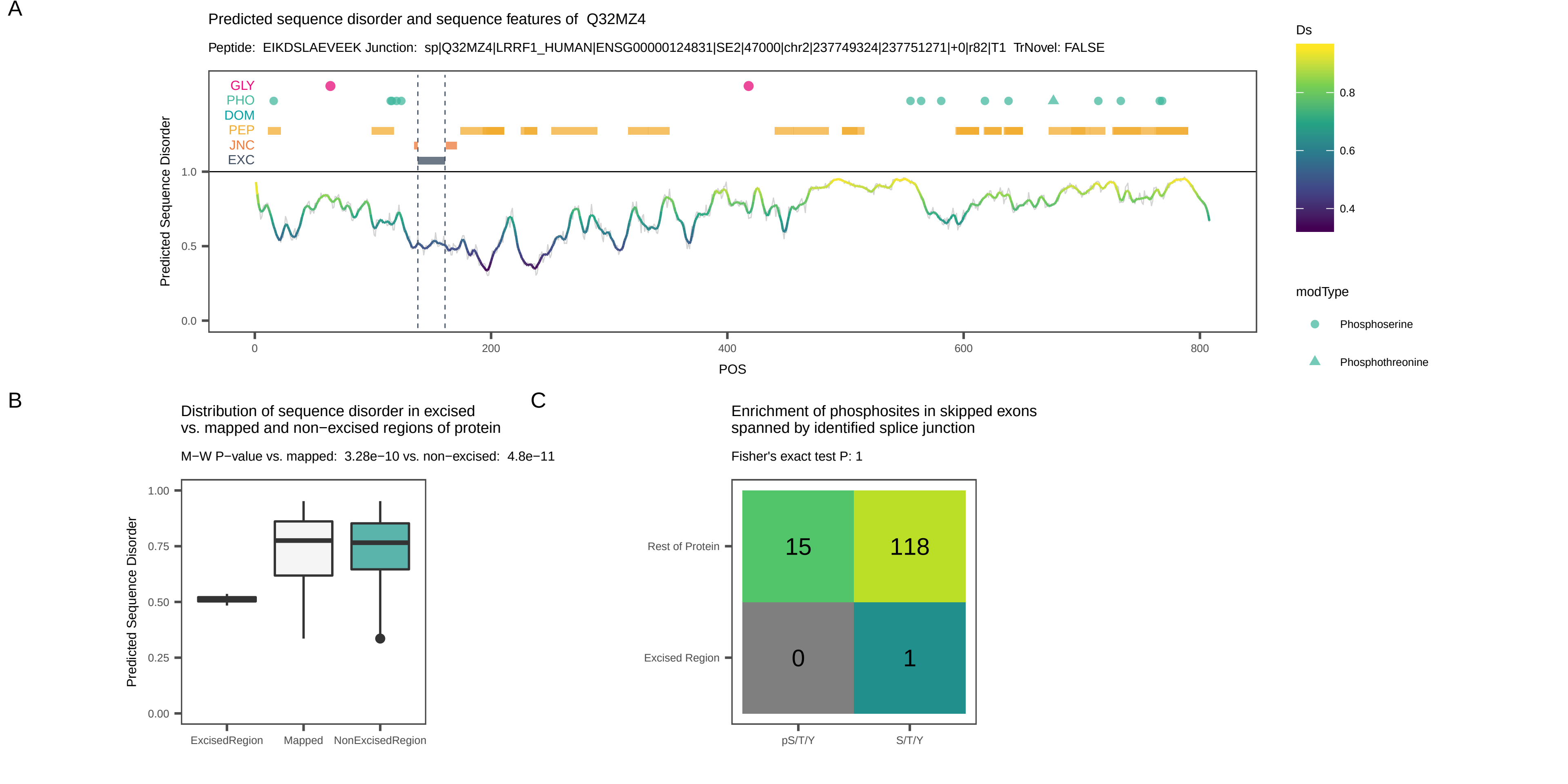
A
Predicted sequence disorder and sequence features of Q32MZ4
Peptide: EIKDSLAEVEEK Junction: sp|Q32MZ4|LRRF1_HUMAN|ENSG00000124831|SE2|47000|chr2|237749324|237751271|+0|r82|T1 TrNovel: FALSE
GLY
PHO
DOM
PEP
JNC
EXC
Predicted Sequence Disorder
1.0
0.5
0.0
0
200
400
600
800
POS
Ds
0.8
0.6
0.4
modType
Phosphoserine
Phosphothreonine
B
Distribution of sequence disorder in excised
vs. mapped and non−excised regions of protein
M−W P−value vs. mapped: 3.28e−10 vs. non−excised: 4.8e−11
Predicted Sequence Disorder
1.00
0.75
0.50
0.25
0.00
ExcisedRegion
Mapped
NonExcisedRegion
C
Enrichment of phosphosites in skipped exons
spanned by identified splice junction
Fisher's exact test P: 1
Rest of Protein
Excised Region
15
118
0
1
pS/T/Y
S/T/Y
